# Letter to the Editor in Response to Gondrand Et al. “Real‐Life Application of a Point‐of‐Care Biosensor for Phenylalanine in Patients With Phenylketonuria”

**DOI:** 10.1002/jimd.70224

**Published:** 2026-07-13

**Authors:** Rachel S. Carling, Emily Whyte, Stuart J. Moat

**Affiliations:** ^1^ Biochemical Sciences, Synnovis Analytics Guy's & St Thomas' NHSFT London UK; ^2^ LGC, the Priestley Centre Surrey Research Park Guildford UK; ^3^ Medical Biochemistry, Immunology & Toxicology University Hospital of Wales Cardiff UK

Dear Editors,

We read with great interest the paper by Gondrand et al. describing the real‐life application of a point‐of‐care biosensor for phenylalanine (PHE) monitoring in phenylketonuria (PKU) [1]. The clinical value of home‐based PHE monitoring is well established, and the burden of frequent laboratory testing on patients and families is substantial. Patients themselves have expressed a clear preference for devices that enable home‐based monitoring solutions [2]. The authors are therefore to be commended for evaluating this promising technology in a real‐life setting. Precisely because of its clinical importance, several analytical and standardisation aspects may benefit from further clarification to ensure the device can fulfil its potential, safely and reliably.

The flow injection analysis tandem mass spectrometry (FIA‐MS/MS) method is described as the “gold standard” throughout; however, this may warrant further qualification. As outlined in reference 7 [3], the method employed represents a semi‐quantitative determination of free PHE in dried blood spots (DBS) commonly used in newborn screening. The limitations of this approach, including imprecision and inaccuracy, have been previously documented [4] and likely contribute to the increasing scatter observed at higher concentrations in Figure 3. We would also welcome clarification as to whether the FIA‐MS/MS DBS results were adjusted to plasma‐equivalent values using the 19% adjustment from reference 7. If so, this represents a comparison between plasma‐adjusted DBS measurements and capillary blood sensor measurements. Such differences in matrix are clinically relevant and should be explicitly acknowledged, as they may introduce systematic bias.

The reported mean positive bias of 84.8 μmol/L is described as ‘small’, yet for children under 12 years the therapeutic target range (120–360 μmol/L) spans just 240 μmol/L. A systematic bias of this magnitude may therefore be clinically relevant and could influence clinical decision‐making, particularly in paediatric management, maternal PKU and determining responsiveness to sapropterin. There are also potential implications for trials with new treatment options. Reagent stability also appears to have a substantial impact on measurement performance. Differences exceeding 400 μmol/L were observed in association with increasing batch age, yet data from batches exceeding the stated shelf life were included in the analysis. Measurements obtained using expired reagents are of uncertain validity and risk obscuring the true analytical performance of the device.

The manuscript states that reagent batches were calibrated using spiked and PKU patient whole blood samples; however, the traceability of these spiked materials and the process used for value‐assignment are not clearly described. For a device intended to support clinical management, calibration should ideally be traceable to a higher‐order certified reference material. In the absence of a certified whole‐blood reference material for PHE, the use of an aqueous certified reference material could provide an external point of reference to support harmonisation and validation of the device.

None of these points detract from the importance of this work. A robustly validated PoC PHE sensor for capillary blood has the potential to eliminate the pre‐analytical variability associated with DBS size and quality [5], which can contribute up to ~40% variability for PHE results, and would meaningfully improve PKU management. Ensuring that results are standardised, or at least harmonised against established methods, will be an essential next step to support safe integration into existing clinical management guidelines. We look forward to seeing this promising work progress.

## Funding

The authors have nothing to report.

## Conflicts of Interest

The authors declare no conflicts of interest.

## Data Availability

Data sharing not applicable to this article as no datasets were generated or analysed during the current study.

## References

[1] C. Gondrand , A. T. Reischl‐Hajiabadi , E. Bonedeau , et al., “Real‐Life Application of a Point‐of‐Care Biosensor for Phenylalanine in Patients With Phenylketonuria,” Journal of Inherited Metabolic Disease 49 (2026): e70187. 41989430 10.1002/jimd.70187 PMC13086054

[2] A. M. Kuypers , K. E. Vliet , A. MacDonald , et al., “Satisfaction With Home Blood Sampling Methods and Expectations for Future Point‐Of‐Care Testing in Phenylketonuria: Perspectives From Patients and Professionals,” Molecular Genetics and Metabolism 142 (2024): 108361. 38442492 10.1016/j.ymgme.2024.108361

[3] D. Haas , J. Hauke , K. V. Schwarz , et al., “Differences of Phenylalanine Concentrations in Dried Blood Spots and in Plasma: Erythrocytes as a Neglected Component for This Observation,” Metabolites 11 (2021): 680. 34677395 10.3390/metabo11100680 PMC8537883

[4] R. S. Carling , Z. Barclay , N. Cantley , et al., “Simple Steps to Achieve Harmonisation and Standardisation of Dried Blood Spot Phenylalanine Measurements and Facilitate Consistent Management of Patients With Phenylketonuria,” Clinical Chemistry and Laboratory Medicine 63 (2025): 1336. 39895045 10.1515/cclm-2024-1367

[5] R. S. George and S. J. Moat , “Effect of Dried Blood Spot Quality on Newborn Screening Analyte Concentrations and Recommendations for Minimum Acceptance Criteria for Sample Analysis,” Clinical Chemistry 62 (2016): 466–475. 26647314 10.1373/clinchem.2015.247668

